# Predicting potential ranges of primary malaria vectors and malaria in northern South America based on projected changes in climate, land cover and human population

**DOI:** 10.1186/s13071-015-1033-9

**Published:** 2015-08-20

**Authors:** Temitope O. Alimi, Douglas O. Fuller, Whitney A. Qualls, Socrates V. Herrera, Myriam Arevalo-Herrera, Martha L. Quinones, Marcus V. G. Lacerda, John C. Beier

**Affiliations:** Abess Center for Ecosystem Science and Policy, University of Miami, Coral Gables, Florida USA; Department of Geography and Regional Studies, University of Miami, Coral Gables, Florida USA; Department of Public Health Sciences, Miller School of Medicine, University of Miami, Miami, Florida USA; Centro de Investigación Científica Caucaseco, Cali, Colombia; School of Health, Valle State University, Cali, Colombia; Department of Public Health, Universidad Nacional de Colombia, Bogota, Colombia; Fundação de Medicina Tropical Dr Heitor Vieira Dourado, Manaus, Amazonas Brazil; Instituto de Pesquisa Leônidas & Maria Deane (FIOCRUZ Amazonas), Manaus, Amazonas Brazil

**Keywords:** Species distribution models, Maxent, Malaria, *An. darlingi*, *An. nuneztovari s.l*, Land-use changes, Climate change, Population expansion, South America

## Abstract

**Background:**

Changes in land use and land cover (LULC) as well as climate are likely to affect the geographic distribution of malaria vectors and parasites in the coming decades. At present, malaria transmission is concentrated mainly in the Amazon basin where extensive agriculture, mining, and logging activities have resulted in changes to local and regional hydrology, massive loss of forest cover, and increased contact between malaria vectors and hosts.

**Methods:**

Employing presence-only records, bioclimatic, topographic, hydrologic, LULC and human population data, we modeled the distribution of malaria and two of its dominant vectors, *Anopheles darlingi*, and *Anopheles nuneztovari s.l.* in northern South America using the species distribution modeling platform Maxent.

**Results:**

Results from our land change modeling indicate that about 70,000 km^2^ of forest land would be lost by 2050 and 78,000 km^2^ by 2070 compared to 2010. The Maxent model predicted zones of relatively high habitat suitability for malaria and the vectors mainly within the Amazon and along coastlines. While areas with malaria are expected to decrease in line with current downward trends, both vectors are predicted to experience range expansions in the future. Elevation, annual precipitation and temperature were influential in all models both current and future. Human population mostly affected *An. darlingi* distribution while LULC changes influenced *An. nuneztovari s.l.* distribution*.*

**Conclusion:**

As the region tackles the challenge of malaria elimination, investigations such as this could be useful for planning and management purposes and aid in predicting and addressing potential impediments to elimination.

**Electronic supplementary material:**

The online version of this article (doi:10.1186/s13071-015-1033-9) contains supplementary material, which is available to authorized users.

## Background

As more countries in Latin America experience economic growth brought about by increased external trade and natural resource exploitation [1], the continuous demand for land to accommodate growing infrastructural development, agricultural and livestock production and frontier settlements, especially in the Amazon, is leading to rapid deforestation [2, 3]. Additional factors such as accelerated rate of internal migrations, expected global climate changes and population expansion [4] may influence the transmission of malaria and other vector-borne diseases [3, 5]. However, the direction of this influence is uncertain; vulnerability to malaria may increase if deforestation continues unabated, or the region could reach a tipping point and experience a major climate shift that may not favor vectors. This could occur as feedbacks between land cover and climate change in the Amazon may lead to rapid (decadal scale) changes within the climate of the Amazon basin itself. For example, recent studies point to the increasing effects of drought in the Amazon on species diversity, biomass, and fires, which appear to be linked to deforestation over the past 30 years [6].

Malaria has persistently plagued Latin America [7], and while there has been marked progress in malaria control in the past decade [8], environmental and population changes potentially threaten these gains by creating conducive habitats [9, 10] and increased availability of blood meals [4] for malaria vectors. Moreover, future temperature changes and ecosystem alterations from local land use patterns may impact malaria transmission by accelerating life cycles of parasites and mosquitoes [11, 12]. At present, our knowledge of both malaria and vector distribution in the region is incomplete [7] although there have been a number of efforts to model the distributions [13–22]. Filling this knowledge gap would help to mitigate potential obstacles to malaria elimination by lending new insights into how vectors and the disease are likely to shift given a business-as-usual scenario.

Malaria risk and the distribution of dominant vectors in Latin America are heterogeneous. Approximately 120 million people in Latin America are at risk of malaria transmission, with an estimated 25 million of them at high risk [8]. Three-quarters of infections are caused by *Plasmodium vivax* (Grassi and Feletti 1890), while *P. falciparum* (Welch 1897) is responsible for the remaining 25 % [8, 23]. The burden of malaria in the region is however borne by countries in the Amazon rainforest in northern South America (NSA) where 90 % of cases are reported [24]. Transmission occurs through infected bites from *Anopheles darlingi* (Root 1926) and *An. nuneztovari s.l.* (Gabaldon 1940), two of the dominant vectors in this region [13]. *Anopheles darlingi* is one of the most efficient and anthropophilic malaria vectors [14], and has been implicated as the primary vector for *P. falciparum* and *P. vivax* in the endemic areas of the region [15, 25]. *Anopheles nuneztovari s.l.* is a species complex in South America comprising of at least two species: *An. nuneztovari* A (from Suriname and Brazil), and *An. nuneztovari* B/C (from Colombia and Venezuela) [26, 27]. *Anopheles nuneztovari* B/C is considered a dominant vector because it bites late into the evening and throughout the night [26], whereas the status of *An. nuneztovari* A as a vector in the Brazilian Amazon is still unresolved [28]. Evidence suggests that both *An. darlingi* and *An. nuneztovari s.l.* are found in altered environments [29–32] and *An. darlingi* prefers locations close to human settlements in frontier agricultural areas in parts of the Amazon [5]. Based on these characteristics and expected bioclimatic changes, an understanding of the distribution of malaria and its vectors, both now and in the future, is needed to aid our preparedness for effective operational malaria control.

A number of attempts have been made to map malaria and vector distribution in the Americas. Gething et al. generated global maps of *P. falciparum* [16] and *P. vivax* [17] endemicity in 2010 using georeferenced parasite rates and incidence data, climatic variables (temperature and aridity) and human population data. Their results showed all nine countries in NSA as having stable or unstable malaria risk [16, 17] and though the Americas accounted for 22 % of global land area at risk, they estimated that the region has 6 % of the global at-risk population for *P. vivax* infection [17].

Previous efforts to map mosquito distributions in the Americas have involved multiple genera [18], or species [13–15, 19, 20] or have been based on single species [22] and at different geographic scales, ranging from continental or sub-continental [13, 14, 18, 22] to national [19, 21]. Foley and colleagues [18] used geo-located museum specimen records to model mosquito species richness and endemicity in the Neotropics. By employing climatic and LULC information, Sinka et al. [13] mapped the distributions of dominant *Anopheles* in the region whereas an eco-regional approach for the Neotropics was used by Rubio-Palis and Zimmerman [20]. Fuller et al. [22] modeled the distribution of *An. albimanus* (Wiedemann 1820) in the Mesoamerican and Caribbean basin based on climatic and topographic data. While some previous attempts have been criticized as lacking a sufficient number of occurrence records and simplicity of techniques used [13], more recent attempts have employed techniques modeling the realized niche or habitat suitability of species. Such studies have generally limited their evaluations of mosquito distribution to bioclimatic, topographic variables [13, 22], and LULC [13]. Moreover, with the exception of Fuller et al. [22], who modeled future distribution of *An. albimanus* by 2080, most studies that have focused on Neotropical vectors have limited their investigations to current distribution patterns. However, the increasing availability of downscaled climate projections from General Circulation Models (GCMs) creates new opportunities to drive modeling techniques that can project future distributions as a function of climate as well as land cover.

Numerous approaches to model species distribution are available, including the presence-only maximum entropy method implemented through the modeling platform, Maxent [33, 34]. Although originally designed to model species habitat suitability, the maximum entropy approach to probabilistic modeling has applications well beyond species niche modeling. For example, some studies have used Maxent to project disease distributions such as dengue [35] and Chagas disease [36]. Thus, using models of this type, one may be able to visualize the current distribution of malaria and where it is likely to persist in the future or shift, and prioritize such areas for current eliminations efforts. In addition, by overlaying current and future distributions, one can visualize where malaria may be continuously problematic through time as a function of climate and land cover change. In this study we model the distribution of malaria, *An. darlingi*, and *An. nuneztovari s.l.* in NSA using bioclimatic, topographic, hydrologic, as well as LULC and population data using Maxent. The aims are to: (i) show the current spatial distribution and examine how the above factors may influence species habitat suitability in NSA and (ii) investigate the potential influence of changes in climate, LULC and population on future species range.

## Methods

**Fig. 1:**
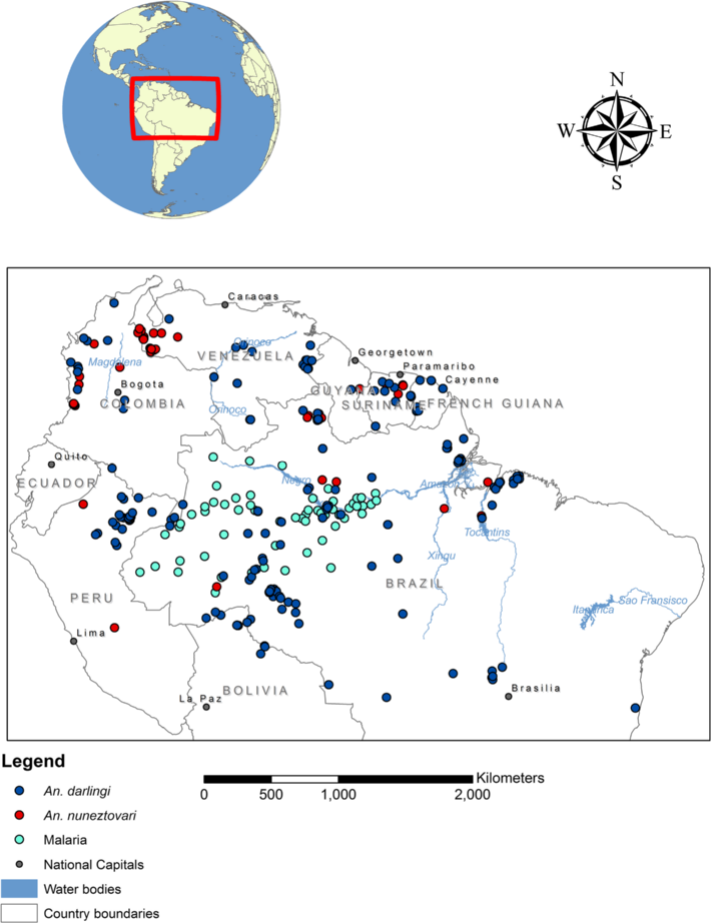
*An. darlingi, An. nuneztovari s.l.* and malaria sample locations. Malaria cases by municipality in Amazonas state of Brazil were converted to population-weighted points representing each municipality

Our study area comprises parts of Bolivia, Brazil, Colombia, Ecuador, French Guiana, Guyana, Peru, Suriname and Venezuela (Fig. 1), and includes all parts of the Amazon rainforest. This area has the combination of socio-environmental and climatic conditions that favor the proliferation of vectors species and malaria.

### Vectors and disease occurrence data

We obtained georeferenced point collection data with records of locations where both larvae and adult *An. darlingi* and *An. nuneztovari s.l.* were sampled (Fig. 1) through VectorMap [37] and the Global Biodiversity Information Facility [38]. These records were collected by different investigators between 1980 and 2007 and made available through an online spatial database, the MosquitoMap [39]. We checked the downloaded data points, excluding those with high estimated spatial uncertainty and multiple entries. Additional sample locations of both species were gathered through field studies conducted in Colombia [40].

*Anopheles darlingi* is present in a wide range in our study area: from the Amazonian south of Venezuela [41, 42], the sparsely populated interiors of Guyana, Suriname and French Guiana [41], to Brazil [15], Amazonian plains of Ecuador [14] and parts of Colombia [43]. It is a lowland, riverine, forest dwelling species whose larvae are often found in lagoons, lakes, slow flowing streams, rivers with shaded clear water, often associated with aquatic macrophytes (AMs) along the shallow margins of water bodies [13, 29, 30, 44]. Larval samples from uncharacteristic locations such as slightly brackish water [29], turbid, polluted water [31], abandoned gold mine dugouts [32] or areas with limited forest cover [5] have also been reported, indicating the influence of environmental changes on its spread. The species is mostly exophilic [25, 32] but exhibits both endophagic [41] and exophagic biting behaviors [45].

*Anopheles nuneztovari s.l.* is found extensively in our study area [46], serving as a primary vector in western Venezuela and north-western Colombia [46], and a probable vector in Suriname [44]. Its larvae are found in both sunlit and shaded habitats in temporary or permanent waterbodies which may contain fresh, clear, still or flowing water with AMs [29, 43]. Samples have also been collected in disturbed habitats such as brick pits and turbid water bodies [29, 31] where this species is readily able to colonize and dominate in disturbed environments [47]. *Anopheles nuneztovari s.l.* is both anthropophilic and zoophilic, rests outdoors [48], and is both exophagic [25, 43, 47–49] and endophilic [48].

Malaria in our study area, caused by both *P. vivax* and *P. falciparum*, is mostly found in Brazil, Colombia, and Venezuela, where 72 % of cases in 2013 occurred [8]. The Brazilian Amazon is the core area where the most malaria infections occur in the region [50]. We therefore obtained malaria incidence data comprising of both parasite species (~75 % of all infections are caused by *P. vivax*) for Amazonas state in Brazil through SIVEP-Malaria (http://200.214.130.44/sivep_malaria/), the national official malaria database. The incidence data originated from passive case detection of patients who reported symptoms consistent with malaria and were cases confirmed using thick-blood smears as is currently the standard procedure in clinics in Brazil. However, we were unable to account for the treatment seeking rates in the area as a review of literature suggests that these are not well established for the Amazon as a whole.

These incidence data were originally aggregated by municipality and then converted to point data using a point-in-polygon analysis in ArcGIS® [51] before being used in the disease distribution modeling (Fig. 1). A population weighting was applied to determine the location of each point representing a municipality, to ensure that the distribution of points were influenced by population clusters rather than being randomly situated in the center of the polygon. Bearing in mind that malaria is usually a rural disease, away from the most densely populated areas, the weighting was carried out such that the points were situated in the least densely populated areas. This was achieved by first creating a fuzzy layer from the population density raster on the premise that populations between 2 and 150 per square kilometer are sufficient for malaria transmission. This fuzzy layer was then converted to points and a spatial join between the points and the municipality polygons was subsequently implemented. The location of the mean center of points was weighted by low population and interpolated from the surrounding points within each municipality. Such weighting was particularly necessary to mitigate limitations of the data. For instance, the aggregation of cases by municipality gave no indication about the exact location of transmission or clustering of cases; however, by locating the points based on population density, a point distribution was achieved.

### Environmental variables

We employed 23 environmental variables as possible explanatory factors in our distribution models. Nineteen bioclimatic variables representing various measures of temperature and precipitation were obtained from WorldClim [52]. This is a set of interpolated global climate surfaces at ~1 km spatial resolution [53]. The layers representing current conditions (1950–2000) and future projections for 2050 and 2070 were collated. The future climate projection layers were chosen from two models from the Intergovernmental Panel on Climate Change (IPCC) Fifth Assessment Report of 2014 [54] - the National Aeronautics and Space Agency (NASA) Goddard Institute for Space Studies (GISS-E2-R) models, and the Hadley Center (HadGEM2-AO) models. The models were chosen because their representation of future predictions of precipitation and temperature in the study area were varied, potentially leading to a range of prediction scenarios. The NASA model generally predicted warmer temperatures compared to the Hadley model in the study area by 2050 and 2070. For precipitation, the Hadley model predicted much drier conditions around the Andes for both periods compared to the NASA models (see Additional file 1), and wetter conditions in parts of the Amazon, and the Atlantic coasts from southern Venezuela to southern Brazil. The NASA model on the other hand predicted higher precipitation in many patches in the Amazon. The climate surfaces were generated under four different greenhouse gas concentration trajectories called representative concentration pathways (RCP). For our analyses, we utilized the most conservative climate projections under the first, RCP 2.6, which assumes a peak in global annual greenhouse gas emissions between 2010–2020, after which emissions are expected to decline [55]. The scenario depicts mean global temperature increase of 1 °C (range from 0.4 to 1.6) by mid-21^st^ century (i.e., 2046–2065) [56].

To account for the altitudinal gradients in the area, which is an important consideration for mosquito and malaria dispersal, we obtained data on elevation from the Shuttle Radar Topographic Mission (SRTM) [57]. An additional factor, the topographic wetness index (TWI) was derived from this topographic information as a measure of soil moisture content especially in low elevation areas [58], providing an indication of potential vector breeding sites. These two layers were gridded to 1 km resolution to retain environmental heterogeneity and ensure data compatibility with other variables. The availability and distribution of human hosts as potential sources of blood meals for vectors was represented using population density layer for 2010 provided by the LandScan product [59]. Since environmental changes, whether natural or human-induced, play an important role in vector and malaria distribution [60], we included changing land use land cover (LULC) patterns in our analysis. The LULC data was derived from Moderate Resolution Imaging Spectrometer (MODIS) imagery for 2001 and 2010 [61], containing 17 LULC classes generated using the International Geosphere-Biosphere Programme (IGBP) classification scheme. The IGBP classes were aggregated into two land cover classes for our land cover projection modeling: forest (containing all forest classes) and non-forested (containing all other classes excluding water bodies).

### Predicting LULC and population changes

To adequately predict distributions of the vectors and malaria for 2050 and 2070, future LULC scenarios as well as population changes in our study area for the requisite periods were projected in Idrisi Selva [62]. The land change modeler (LCM) was used to estimate LULC changes. LCM is an application designed to model land conversion by using historical changes from land cover maps to project future land use change scenarios [63]. The process began with the introduction of land cover maps of the two time periods, 2001 and 2010 to assess changes between them. By incorporating change drivers related to forest access such as distance from roads, water bodies [64], past deforestation [61], and elevation [57], land use transition potentials were produced. Probability of change (transition probabilities) between both time periods was quantified using the Markov transition matrix [65]. We assumed the transition probabilities remain unchanged over time, and used these to project future LULC scenarios for 2050 and 2070. LULC change was estimated using the Area module in Idrisi and each land cover map for 2010, 2050 and 2070 entered as a categorical variable in Maxent for the species distribution modeling for the respective time periods.

Population changes for 2050 and 2070 were predicted by applying an exponential population function (Equation 1) to the base population year, 2010. Using an average annual growth rate of 1.1 % across the region [66], the projected population for the two time periods was estimated by the formula:

1 $$ \mathrm{P}\kern0.5em =\kern0.5em {\mathrm{P}}_0\kern0.5em \cdotp \kern0.5em {\mathrm{e}}^{\mathrm{rt}} $$

Where P = Estimated population, P_0_ = Initial population, r = rate of natural increase, and t = number of years between P and P_0_.

### Modeling species and malaria distributions using maximum entropy

Maxent is a machine-learning method that estimates the distribution of a target by finding the distribution with the largest spread [67]. Maxent predicts species habitat suitability by incorporating its documented occurrence points with relevant environmental predictors in a defined geographic space [68, 69], subject to the constraint that the expected value of each predictor under this estimated distribution matches its empirical average [70]. The output is the relative habitat suitability calculated by converting the exponential values of the raw estimates of suitability to logistic values [70].

All cleaned presence data for *An. darlingi* (*n* = 271), *An. nuneztovari s.l.* (*n* = 175) and Malaria (*n* = 62) (Fig. 1) were used in the distribution models. Twenty-five percent of each of the datasets were randomly selected to independently assess the accuracy of each model while the rest were used for model training. The collection data used were suited for our analyses at ~1 km resolution because the distance between the data points were relatively greater than 1 km. The resolution was selected to be consistent with the highest resolution available for the environmental layers as has been used by other studies e.g., Drake and Beier, 2014 [71].

Due to a lack of absence data, a set of pseudo-absence (background) points were created for each species as Maxent determines habitat suitability by relating the values of predictors at presence points with those of randomly generated pseudo-absence points within the same area [72, 73]. Knowing that the number of pseudo-absences affects the models [13], we created multiple background points in ratios 1:1, 2:1, 5:1, and 10:1 to presence points for the vectors and evaluated the model results both visually (mapped distribution) and statistically (how well the area under the curve (AUC) improved). Based on the evaluation, the optimal ratio of background to presence points was 1:1 for the vectors while the default 10, 000 background points in Maxent was optimal for malaria. To account for the inherent sampling bias in the presence data [69], bias files encompassing the area of study were created for each species to ensure that both the occurrence and pseudo-absence points had the same geographical bias [74]. We tested for multi-collinearity among pairs of predictors and excluded one predictor in each pair that showed high correlation (Table 1) in the vector models. However all 23 predictors were used in the malaria model as AUC value improved when this was done.

**Table 1:** MaxEnt models validation parameters evaluated using test points

Species	Time/Model	Parameters in model	Training AUC	Test AUC	Mean {sd}^b^	ETSS	Omission rate	TSS
Malaria	Current	23	0.93	0.9	0.53 {0.23}	0.271	0.25*	0.57
	2050 (Hadley)				0.49 {0.16}			
	2050 (NASA)				0.46 {0.18}			
	2070 (Hadley)				0.46 {0.17}			
	2070 (NASA)				0.45 {0.19}			
*An. darlingi* ^a^	Current	13	0.77	0.75	0.51 {0.12}	0.463	0.34*	0.58
	2050 (Hadley)				0.51 {0.14}			
	2050 (NASA)				0.50 {0.13}			
	2070 (Hadley)				0.51 {0.13}			
	2070 (NASA)				0.51 {0.13}			
*An. nuneztovari s.l.* ^a^	Current		0.8	0.79	0.53 {0.14}	0.492	0.3*	0.68
	2050 (Hadley)				0.53 {0.12}			
	2050 (NASA)				0.55 {0.10}			
	2070 (Hadley)				0.54 {0.11}			
	2070 (NASA)				0.53 {0.13}			

*Significant at *p* < 0.001

^a^Excluded parameters with high correlation to avoid over-fitting

^b^Estimated using 12, 65 and 44 test and background points each for Malaria, *An. darlingi* and *An. nuneztovari* respectively

Modeling was carried out by identifying the current niche suitability drivers from the current models and based on the assumption that all factors remained constant except a changing landscape, we applied these to the future scenarios. We used auto features for model generation, an option which allowed the set of features used to be determined by the number of presence points, using general empirically-derived rules. All modeling was performed using the subsample replicated run in Maxent v 3.3.3 k, the most current version of the software [75].

### Assessing model performance

Various measures of model performance were used in our accuracy assessment. The model’s ability to discriminate between species presence and absence sites was measured using AUC [76]. Generally, models with AUC ≤ 0.5 are deemed to behave no better than random, while an AUC of 1 indicates a perfect fit between observed and predicted surfaces [77]. In practice, models with AUC above 0.75 are considered useful and results applicable [70]. Further assessment of model performance requires setting a threshold at which species habitat suitability can be converted to binary predictions of species presence or absence [78, 79]. While many threshold approaches are available, we chose the sensitivity-specificity equality approach that has been identified as one of the best performing thresholds [78]. The equal sensitivity- specificity threshold (ETSS) maximizes the absolute value of the difference between sensitivity and specificity [78, 80]. A good model is expected to accurately predict a high proportion of test sites by having a low omission rate or high sensitivity [81]. However, because it possible for a model to have high sensitivity (low omission rate) just by predicting species presence in large parts of the area of interest, we evaluated the statistical significance of the omission rate obtained using the exact one-tailed binomial test (because of the small size of the test data) [82, 83]. The acceptability of the omission rate is determined by comparing the observed rate to theoretical expectations [84]. For instance, ideally in a model where the threshold is the 10^th^ percentile presence, approximately 10% omission is theoretically expected. Omission rates higher than this value therefore indicate overfitting [84]. In our study, we used the ETSS as our threshold, therefore omission rates less than the ETSS value for each model are acceptable. Finally, because the AUC has been criticized as assessing the degree to which predictors can restrict species range rather than model performance [83, 85], we employed the true skill statistic (TSS) as a further test of model performance. The true skill statistic is the mean of net prediction success rate for presence and absence [83, 86]. Although TSS takes into account omission and commission errors, it avoids reliance on prevalence or size of validation set, and is thus a good measure of predictive accuracy of presence-only models [85]. TSS values also range from 0 to 1, with values >0.6 considered good, >0.7 very good [87].

### Limitations

Although modeling *P. falciparum* and *P. vivax* distributions separately may have been more informative, with the possibility of directing interventions specific to each parasite species [88], our data access was limited to pooled malaria data in which the infections were not distinctly identified. However, of the two parasites, modeling *P. vivax* distribution may have been more arduous given its latent hypnozoite stage [88], which may be difficult to account for in our models, especially for the future scenarios. We also assumed a constant rate of deforestation in our LULC model and that deforestation and climate change are independent, which may be unrealistic given the number of studies that have linked deforestation with global and regional climate change [89, 90]. Finally, we had no information on detection probability, i.e., the probability of a species being detected given that it occupies a location (occurrence probability) and that sampling was conducted in that location [91]. This probability often varies with the same covariates that determine occurrence probability [92], and when not separated from occurrence probability, may under- or over-estimate model results [92]. Thus, we ask readers to exercise caution in interpreting model results.

## Results

### LULC changes

The LCM outputs for LULC changes in 2050 and 2070 are presented in Additional file 2. The maps indicate that at current rates of deforestation, large parts of the Amazon forest, particularly in the interior and the South would be lost by the mid-century, assuming a business-as-usual scenario in which deforestation progresses at approximately the same rate as over the past decade. Most of the loss is expected along transportation routes (roads and rivers) as the interior opens up to urbanization and infrastructural developments. Forest loss would also increase in the South, particularly in Beni and Santa Cruz (Bolivia), Mato Grosso, Rondônia, Pará, Maranhão and Tocantins (in Brazil, particularly due to soy plantations), along the coasts of Guyana, French Guiana and Suriname. Non-forest areas, such as the savanna between Roraima (Brazil) and Bolivar (Venezuela) are also expected to expand by 2050 and 2070. While our results are similar to those previously published [93], we advise caution in the interpretation as the change pattern in the interior closely follows the drivers of LULC change we employed. Altogether, our forecast indicates an estimated 780,000 km^2^ of forests would be lost area by 2070 (Table 2), a development that may have an impact on vector and malaria distribution in the region.

**Table 2:** Summary statistics of projected changes in LULC

Year	Category (Km^2^)		Period	Change (Km^2^)
	Forested	Deforested		
2010	6432680	5903650	2010–2050	−699475
2050	5733205	6603125	2010–2070	−777265
2070	5655415	6680915	2050–2070	−77790

### Habitat suitability modeled using current conditions

**Fig. 2:**
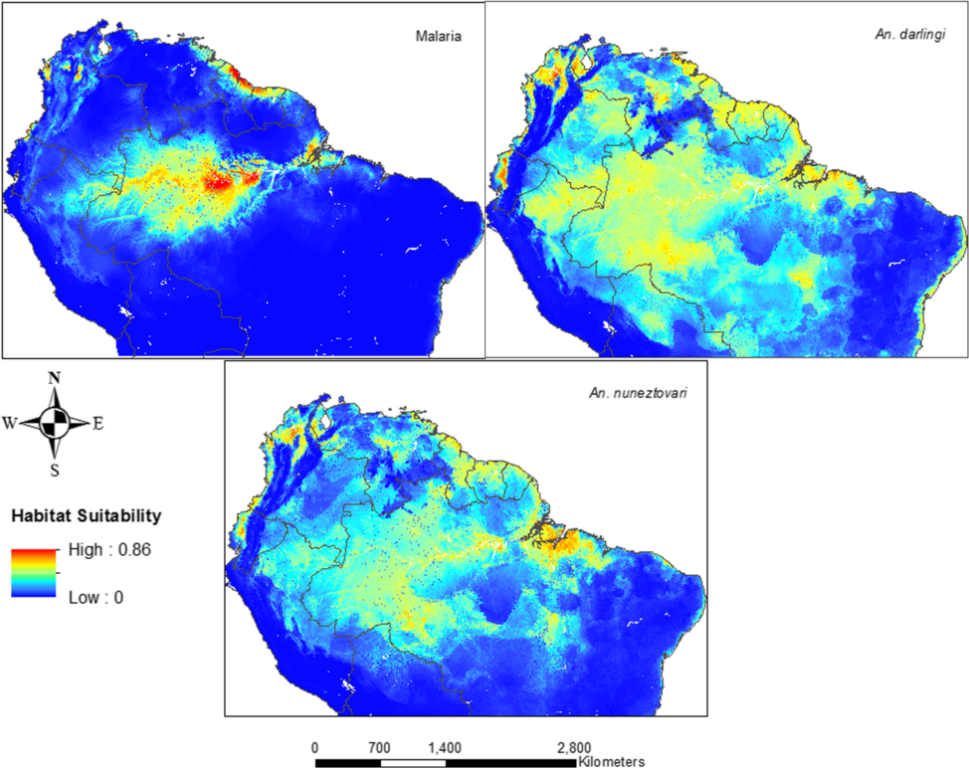
Habitat suitability for Malaria (top left), An. nuneztovari s.l. (top right), and An. darlingi (bottom panel) modeled using current bioclimatic conditions, population, LULC, elevation and TWI

Predictive maps of habitat suitability using current conditions are presented in Fig. 2. For malaria, areas of relatively high habitat suitability (high = 0.5–0.75; very high 0.75–1) are predicted within the interior of the Amazon in Brazil, along the coasts of the Guianas, along the Pacific coast of Colombia and in western Venezuela, covering a total land area of about 672,000 km^2^. Zones of moderate habitat suitability (0.25–0.5) are predicted in the Amazonian regions of Peru, Bolivia and Colombia while the rest of the study area, including the Andes and the Brazilian highlands have low habitat suitability (<0.25). Elevation was the biggest contributor to the model (45.6 %), followed by precipitation of the driest quarter (16.3 %), mean temperature of the coldest month (12.9 %) and precipitation of the driest month (9 %). Population (0.1 %) and LULC (0.2 %) did not contribute to the model (Additional file 3 for response curves and jackknife of variable importance). The model had excellent discriminatory power as indicated by the test AUC (0.90) (Table 1). The mean values of the test points and ETSS were relatively moderate, as was the omission rate showing that a reasonable proportion of the test sites were correctly predicted. The reported TSS value of 0.57 also showed a fair indication of the model performance.

Model results for *An. nuneztovari s.l.* show areas of relatively high habitat suitability along the coast of Ecuador, the Pacific coast and the Llanos of Colombia, western Venezuela, the coasts in the Guiana Shield, and the states of Pará, Mato Grosso and Amazonas in Brazil, occupying about 460, 000 km^2^ of land area. Relatively moderate to low (<0.5) suitability are shown in other parts of Colombia, Peru, Bolivia, Venezuela and the rest of Brazil. As expected, the presence of this vector is not predicted in the Andes where high altitudes prevail. Elevation was the most important predictor for this species (33.4 %), followed closely by temperature seasonality (30 %) and annual precipitation (13.9 %). Population (8.7 %) and LULC (4.4 %) also contributed marginally to the model (Response curves in Additional file 4). The surface depicting *An. nuneztovari s.l.* habitat suitability (Fig. 2) clearly distinguished between presence and absence sites (AUC = 0.79), had a good TSS value (0.68) and a moderate omission rate (0.30) (Table 1).

Relatively high suitability were reported for *An. darlingi* in areas such as the interior of Ecuador, the Pacific and Caribbean coasts of Colombia, the Llanos, western and coastal Venezuela, the coasts of the Guianas, the Amazonian states in Brazil and Loreto in Peru, an area of approximately 920,000 km^2^. Moderate suitability was predicted in the other parts of the study area except the Andean mountains, the Brazilian Highlands and a few patches within the Amazon where probabilities are low. Elevation alone accounted for 53.3 % of the model while annual precipitation (18.4 %) and population (11 %) were the next biggest contributors. Precipitation seasonality (7.9 %) was a marginal contributor whereas LULC did not influence the model (Additional file 5). This suggests a limitation of the Maxent model rather than lack of influence from land cover and land use, as Fuller et al. [65] found that Maxent sometimes produced unrealistic results when categorical land cover maps were used as covariates. This model had fair discriminatory power (AUC = 0.75), a fair TSS value (0.58) and moderate omission rate.

### Habitat suitability modeled using predicted future conditions

#### Malaria

**Fig. 3:**
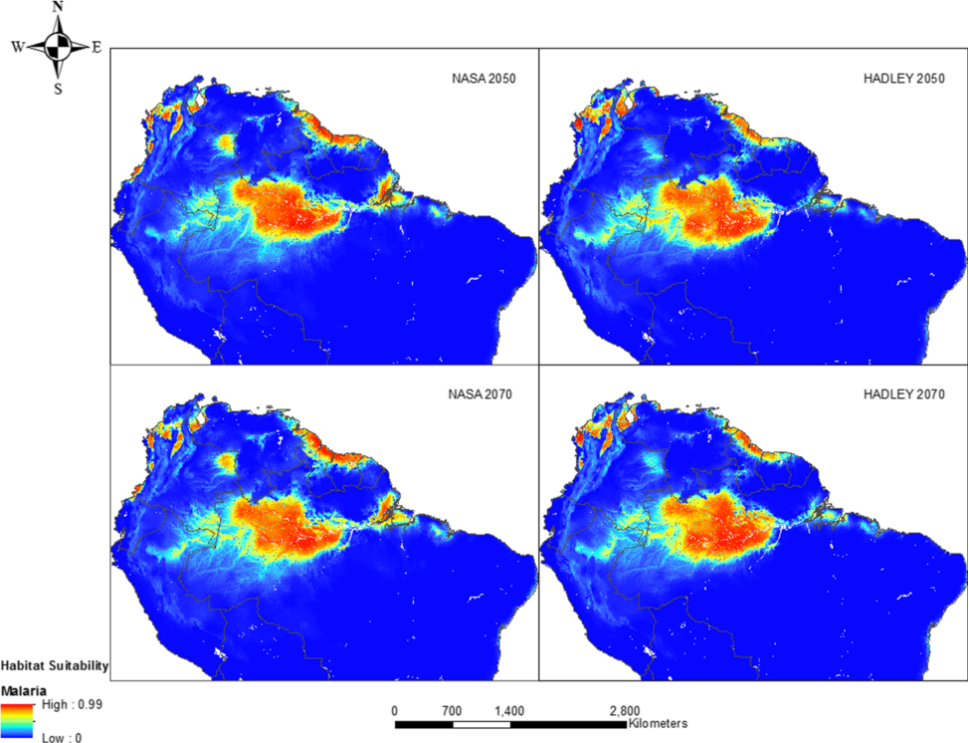
Habitat suitability for malaria modeled using future climate, population and LULC: NASA 2050 (top left), 2070 (bottom left) and Hadley 2050 (top right), 2070 (bottom right)

Figure 3 reveals the projected distributions for malaria in the years 2050 and 2070 using the NASA and Hadley center climate models. As shown, the foci of malaria are expected to remain in the interior of the Amazon, along the coasts in the Guiana Shield, in northern Colombia and along the southern border of Colombia and Venezuela. Moderate habitat suitability (0.25–0.5) was predicted mostly around North-western Brazil, eastern Peru and South-western Colombia in the NASA model while the Hadley model prediction included more regions in the Amazon and Bolivia. However, total land area of suitability is expected to decrease compared with current distributions, except with the 2050 Hadley model. By 2050, the NASA model predicts a 28 % reduction in suitable area whereas a 3 % increase is estimated from the Hadley center model.

By 2070 however, the area is expected to have decreased by 6 and 17 % according to the Hadley and NASA models, respectively, compared with the current distribution. The NASA model is less conservative, predicting lesser areas of malaria presence by 2050 and 2070, but bigger changes. When the gains and losses in each habitat suitability category were analyzed for the future predictions (see Additional file 6), only areas of low suitability reduced in area (~129,000 km^2^) between 2050 and 2070 whereas the other categories gained in the NASA model. With the Hadley model, areas of low and very high suitability increased in area (~33,000 km^2^ and 27,000 km^2^ respectively) while medium and high suitability had losses. The full cross-tabulation of current and future distributions of malaria is presented in (Additional file 7). Maxent predicted a range contraction within the Amazon, in the coast of Guyana, Antioquia and Choco in Colombia, and around the northern border between Colombia and Venezuela. Range expansion was predicted around the border of Brazil, Colombia and Peru and in south-east Colombia.

#### Anopheles nuneztovari s.l.

**Fig. 4:**
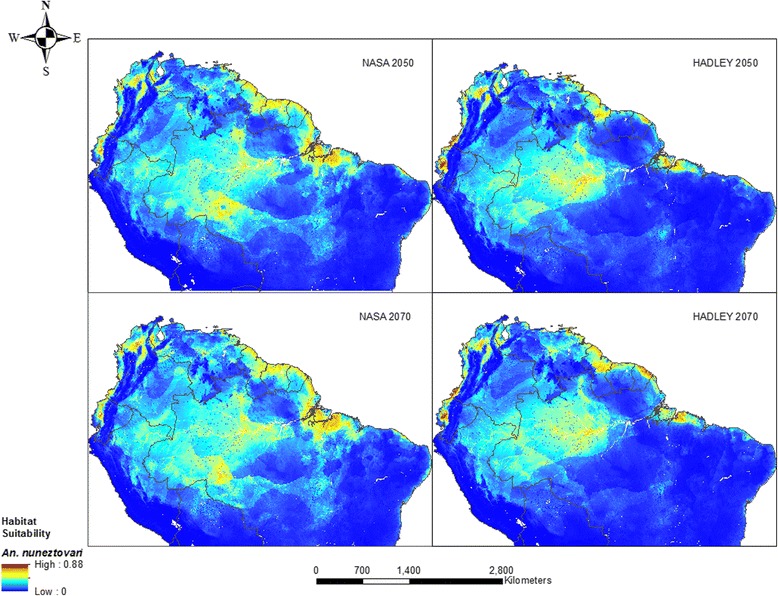
Habitat suitability for *An. nuneztovari s.l.* modeled using future climate, population and LULC: NASA 2050 (top left), 2070 (bottom left) and Hadley 2050 (top right), 2070 (bottom right)

Areas of relatively high habitat suitability for *An. nuneztovari s.l.* are predicted along rivers in the Amazon, the coasts in the eastern part of the study area, and in patches in Venezuela and Colombia from both models for 2050 (Fig. 4). Most of the Amazon, eastern Brazil and middle belt of Colombia and Venezuela have moderate probabilities of vector presence. Both the Hadley and NASA models forecast a 5 and 20 % increase in range respectively by 2050. It is noteworthy that though the species was mostly absent around the Andes with current conditions, its presence in this area is predicted by 2050. The range is expected to increase by 14 % in 2070 according to the Hadley model whereas a 10 % increase is projected by NASA model. During this period, the Llanos, southern Colombia, eastern Peru, Pacific and Caribbean coasts of Colombia and large parts of the Amazon have medium predicted probabilities as estimated by the Hadley model whereas most of the Llanos are excluded in the NASA model.

As with the malaria model, only areas of low suitability in the NASA model decreased in size (~232,100 km^2^) when gains and losses were analyzed between the 2050 and 2070 models. On the other hand, moderate and high suitability areas gained, while low and very high suitability areas lost in the Hadley model for the same period (Additional file 6). *An. nuneztovari s.l.* range is shown to expand in the Amazon interior possibly along transportation routes, and along the coast in the Guianas according to NASA model (see Additional file 8). A similar pattern is observed along the coast in the Hadley model with additional areas in parts of Brazil and at the border of Venezuela and Colombia. Range contraction is mostly expected around Pará in Brazil, along the Pacific coast of Ecuador and Colombia, and in northern Colombia by both periods.

#### Anopheles darlingi

**Fig. 5:**
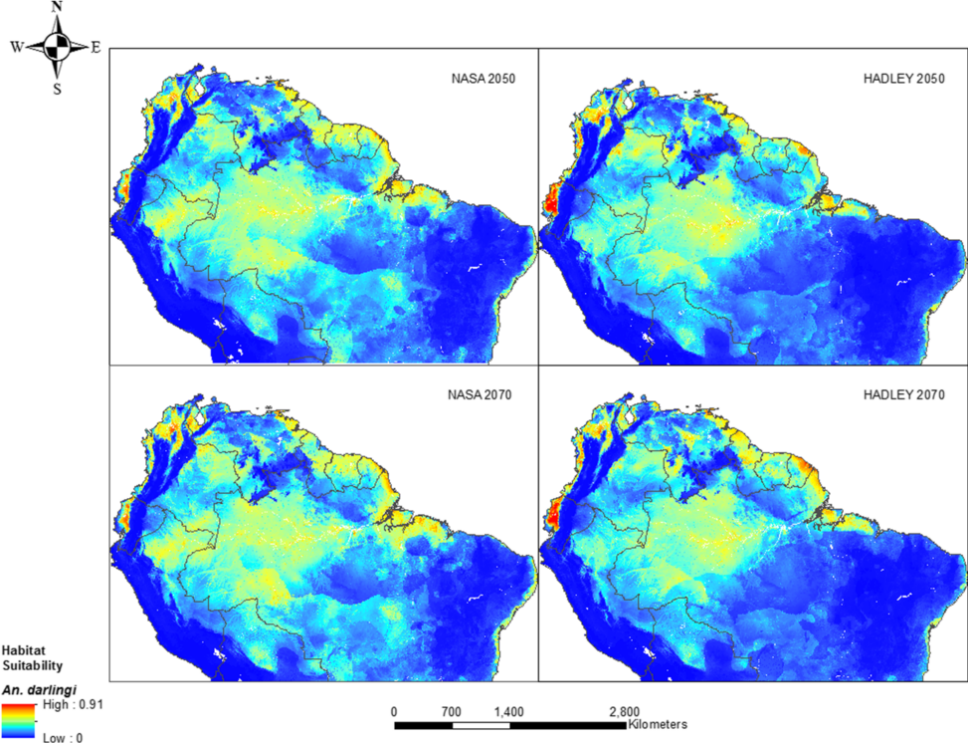
Habitat suitability for *An. darlingi* modeled using future climate, population and LULC: NASA 2050 (top left), 2070 (bottom left) and Hadley 2050 (top right), 2070 (bottom right)

Most parts of the study area are expected to remain favorable to *An. darlingi* presence by 2050 and 2070 according to model predictions (Fig. 5). Areas of relatively high suitability are mostly found along the coasts in Ecuador, Colombia, the Guianas and Brazil. Other patches are in the Amazonian regions of Colombia, Peru and Brazil whereas moderate suitability was estimated mainly in the Amazon, Venezuela and the Guianas. While the Hadley model predicts a slight reduction in land area by 2050 and 2070, the NASA model estimates no decline in range by 2050 but a 3 % increase by 2070.

Again, areas of moderate, high and very high suitability gained in area between 2050 and 2070 in the NASA model whereas only low suitability areas (~107,000 km^2^) gained in the Hadley model for same period (see Additional file 6). Range contraction is predicted for *An. darlingi* in patches around Rondônia, the Amazon states in Brazil, Colombia and Peru, and Apure and Bolivar in Venezuela (Additional file 9). Expansion is expected in a few areas around Bolivia, Brazil, Colombia and the Guianas.

## Discussion

Our study is unique in that it investigates the influence of climate, LULC and population changes on potential distributions of the malaria parasites, *An. darlingi*, and *An. nuneztovari s.l.* in NSA. We applied the presence-only Maxent model to project the current and future spatial distribution of malaria parasites and mosquito vector species, highlighting the potential environmental drivers of changes in their ranges. The relatively moderate-to-high AUC values for *An. darlingi*, malaria and *An. nuneztovari s.l.* respectively not only reveal the model’s ability to distinguish presence and absence sites [76], but also the higher chance of occurrence points being given relatively higher probabilities of presence compared to pseudo-absence points [73]. The TSS values reported also indicate that our models were fairly accurate in predicting the presence and absence of the species by keeping false positives and negatives to a minimum [83]. When the ability of the models to predict test sites was evaluated, they were relatively sensitive with significantly moderate omission rates, indicating that many of the test points fall into areas predicted present by the models [81, 82], even as the mean probabilities of the test points were moderate as well.

The maps produced based on current conditions for both vectors and malaria parasites to a large extent agree with other studies. Our modeled potential distribution of *An. darlingi* and *An. nuneztovari s.l.* are consistent with previously published work by Sinka et al. [13], except for areas of slight divergence around the Brazilian Highlands, and parts of the Andes and the Pacific coast in Ecuador and Colombia for *An. darlingi* and *An. nuneztovari s.l*. The malaria map differs from earlier published extent of endemicity by Gething et al. [16, 17] mostly because our occurrence points are from one state within the Amazon and combine both parasites. However, the model accurately predicts core areas of high malaria incidence within the sub-region where control efforts should be focused. Moreover, current malaria interventions such as vector control were not considered in the model development. Thus, taking into account the possibility of anophelism without malaria (i.e., the occurrence of *Anopheles* vectors in an area/region without malaria [94, 95] as was discovered in Europe for the species complex, *An. maculipennis* [96] and may be the case with *An. nuneztovari* species complex in the Brazilian Amazon [28]), it is highly plausible that the actual extent of malaria is limited to areas of known incidence in the region, rather than where vectors may be found. Moreover, a map depicting probability of vector presence does not necessarily imply risk of the parasites it transmits [95].

When projected on future climatic and human-induced changes, model simulations generally showed a decrease in malaria extent by 2050 and 2070. The areas of range contraction for malaria (in Brazil, Guyana and Colombia) in particular bolster optimism as these are currently the localities with some of the highest malaria incidence in the region [12, 97, 98]. These results are especially informative when considering the renewed drive towards malaria elimination in the region [99]. Although the area extent for the vectors are projected to increase, the decrease in malaria extent despite this implies that the interplay of climatic, population and local land use patterns can naturally force a decline in malaria incidence [71]. For example, development may lead to lower malaria as more infrastructure and better living conditions become available, while climate change and deforestation produce range expansion of vectors. The spatial extent of malaria may decrease even further as principles of integrated vector management (IVM) become more entrenched in vector control programs [100], surveillance and monitoring are sustained, more efficient and effective drugs and vector control measures become available, and malaria treatment become more accessible [7]. Elimination in this region by 2050 may be feasible as strategies outlined by Feacham [101], such as development of new drugs, vaccines, and insecticides and strengthening national and regional collaborations are executed. Unsurprisingly, measures of precipitation and temperature as well as elevation were the highest predictors of malaria in the region as found in several previous studies [102–104]. The impacts are especially important as other studies have shown that climate change leads to warmer and drier conditions, which may aid mosquito and parasite development, and thus potentially increase malaria risk, even in highland areas [9].

Future projections reveal a modest increase for *An. darlingi* and a slightly larger range expansion for *An. nuneztovari s.l.* by 2050 and 2070. The areas of range expansion and contraction for both species are likely to be influenced by human activities as more parts of the Amazon become urbanized, infrastructural projects increase [12] and gold mining continues [105]. This is especially important as change in LULC was a predictor for *An. nuneztovari s.l.*, consistent with earlier reports of the species colonizing altered environments and being associated with deforestation [29–32, 98]. Surprisingly, LULC was not a predictor for *An. darlingi* in our models despite numerous studies indicating a correlation between the species and land cover or deforestation [9, 10, 12, 29–32, 98]. Population density was shown as an important predictor for *An. darlingi* and a marginal predictor for *An. nuneztovari s.l.*, consistent with their known behavior [5, 9] and that of other species such as *Culex pipiens* (Linnaeus 1758) [73]. Elevation was the most important predictor of both species and malaria for all models. Such a result was expected considering that recent studies have reported some other mosquito species at higher altitudes than regularly found, for example in Ecuador [106] and projected for parts of Mesoamerica in 2080 [22]. Moreover, malaria has been reported in highland areas of Bolivia [107] and some East-African countries [102, 108, 109], so vectors must be present for transmission. Finally, our results are in agreement with the current understanding of climate interaction with mosquitoes; i.e., temperature and precipitation were major contributors to the projected vector distributions for all climate scenarios. This is supported by other studies that have established associations between temperature, precipitation and both *An. darlingi* and *An. nuneztovari s.l.* [12, 13, 98]. These variables have also been linked to other *Anopheles* species [102, 110–112], and to *An. albimanus* [22] and *An. arabiensis* [65, 71] in future periods.

## Conclusion

We presented models of the current and future spatial distribution of malaria, *An. darlingi*, and *An. nuneztovari s.l.* in NSA using bioclimatic, topographic, hydrologic, LULC and population data. Our analyses reveal that while climatic factors, temperature and precipitation, play important roles in current and future distribution of malaria parasites, *An. darlingi*, and *An. nuneztovari s.l.* in the region, aspects of human influence measured by LULC and population changes will also affect the distribution of *An. nuneztovari s.l.* and *An. darlingi* respectively. As such, stricter regulations need to be enforced and sustained to reduce further deforestation in the Amazon. Although the models project increased range for the vectors, sustained vector control as well as deployment of novel strategies in the near future could prevent this expansion. Based on the factors analyzed, malaria extent is expected to naturally decrease in the future. Thus, with increased implementation of IVM strategies and more effective anti-malaria drugs, trajectories of climate change and deforestation may complement efforts underway to achieve the goal of malaria elimination in NSA in the coming decades.

## Additional files

Additional file 1:
**Differences in annual precipitation between current and future conditions.** NASA 2050 (top left), 2070 (bottom left) and Hadley 2050 (top right), 2070 (bottom right). (TIFF 24678 kb) Additional file 2:
**Projected LULC changes obtained using LCM: 2010 (top panel), 2050 (middle panel), and 2070 (bottom panel).** (TIFF 24675 kb) Additional file 3:
**Response curves of Malaria model to: (A) Elevation (srtm); (B) Precipitation of the driest quarter (bio 17); (C) Mean temperature of the coldest month (bio 6); (D) Precipitation of the driest month (bio 14); and (E) Jacknife of variable importance (Red bar shows the gain when all variables are used.** The light blue bar shows the gain when a specific variable is excluded from analysis, a lower gain indicating that the specific variable has more information not contained in other variables. The dark blue bar indicates gain when the specific variable is used in isolation). (PDF 257 kb) Additional file 4:
**Response curves of** ***An. nuneztovari*** **model to: (A) Elevation (srtm); (B) Temperature seasonality (bio 4); (C) Annual precipitation (bio 12); (D) Population; (E) LULC; and (F) Jacknife of variable importance (Red bar shows the gain when all variables are used.** The light blue bar shows the gain when a specific variable is excluded from analysis, a lower gain indicating that the specific variable has more information not contained in other variables. The dark blue bar indicates gain when the specific variable is used in isolation) (PDF 258 kb) Additional file 5:
**Response curves of** ***An. darlingi*** **model to: (A) Elevation (srtm); (B) Annual precipitation (bio 12); (C) Population; (D) Precipitation seasonality (bio 15); and (E) Jacknife of variable importance (Red bar shows the gain when all variables are used.** The light blue bar shows the gain when a specific variable is excluded from analysis, a lower gain indicating that the specific variable has more information not contained in other variables. The dark blue bar indicates gain when the specific variable is used in isolation). (PDF 255 kb) Additional file 6:
**Analysis of gains and losses in area between 2050 and 2070 models.** From left to right: Malaria (NASA (top), Hadley (bottom)), *An. darlingi* (NASA (top), Hadley (bottom)), *An. nuneztovari* (NASA (top), Hadley (bottom)). (TIFF 310086 kb) Additional file 7:
**Cross-tabulation of present and future distribution to show likely shifts in Malaria habitat suitability: NASA 2050 (top left), 2070 (bottom left) and Hadley 2050 (top right), 2070 (bottom right).** (TIFF 2525 kb) Additional file 8:
**Cross-tabulation of present and future distribution to show likely shifts in** ***An. nuneztovari s.l.*** **habitat suitability: NASA 2050 (top left), 2070 (bottom left) and Hadley 2050 (top right), 2070 (bottom right).** (TIFF 24676 kb) Additional file 9:
**Cross-tabulation of present and future distribution to show likely shifts in** ***An. darlingi*** **habitat suitability:** **NASA 2050 (top left), 2070 (bottom left) and Hadley 2050 (top right), 2070 (bottom right).** (TIFF 24677 kb)

## Abbreviations

LULC: Land use and land cover
NSA: northern South America
SDM: Species distribution models
GCM: General circulation models
IPCC: Intergovernmental panel on climate change
NASA: National Aeronautics and Space Agency
GISS: Goddard Institute for Space Studies
RCP: Representative concentration pathways
SRTM: Shuttle Radar Topographic Mission
TWI: Topographic wetness index
MODIS: Moderate Resolution Imaging Spectrometer
IGBP: International Geosphere-Biosphere Programme
LCM: Land change modeler
AUC: Area under the curve
TSS: True skill statistic
IVM: Integrated vector management
