# Supplementary figures and images for: Predicting potential ranges of primary malaria vectors and malaria in northern South America based on projected changes in climate, land cover and human population
Source: Parasit Vectors. 2015 Aug 20;8:431. doi: 10.1186/s13071-015-1033-9 (PMC4546039; doi:10.1186/s13071-015-1033-9)

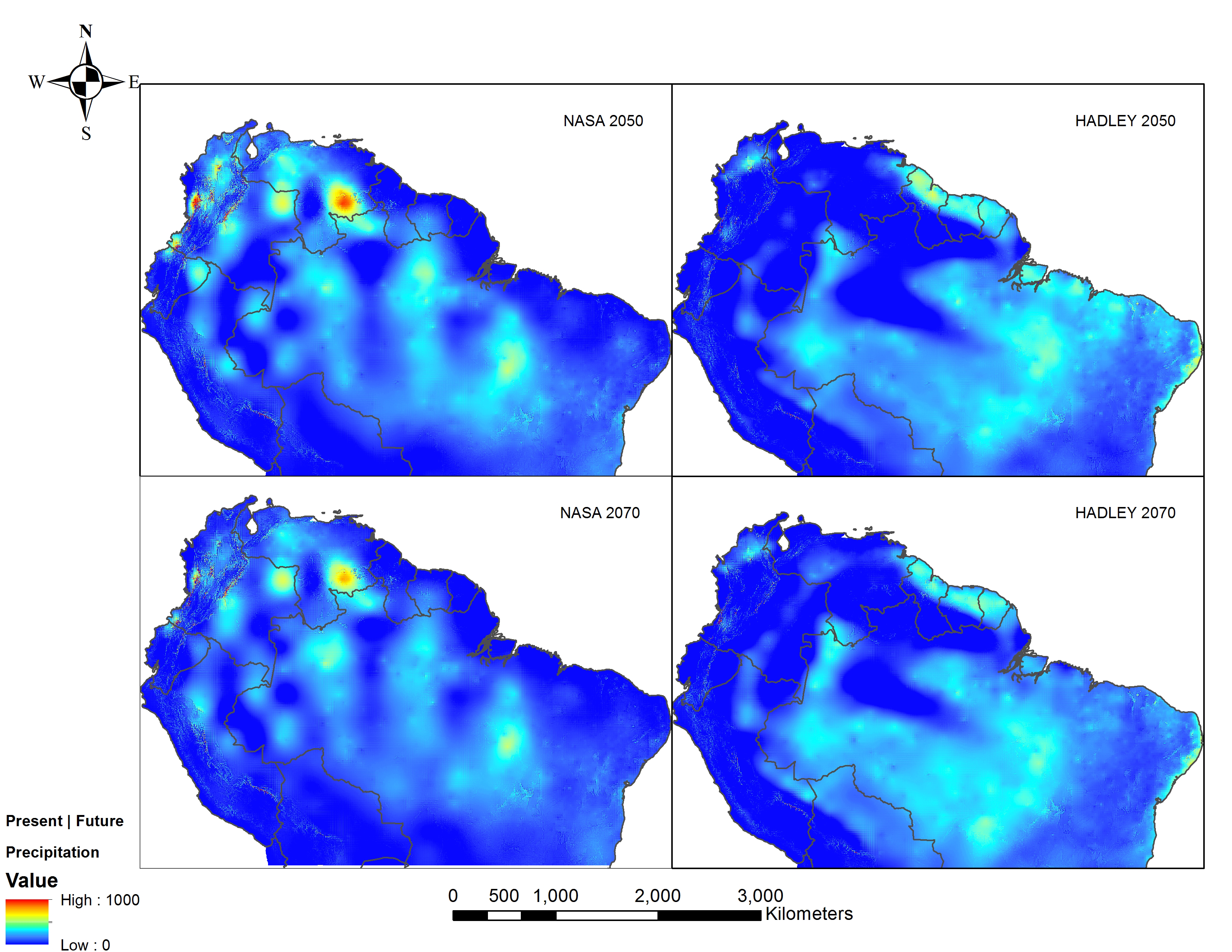

Supplement: Additional file 1: — Differences in annual precipitation between current and future conditions. NASA 2050 (top left), 2070 (bottom left) and Hadley 2050 (top right), 2070 (bottom right). (TIFF 24678 kb) [file 13071_2015_1033_MOESM1_ESM.tif]

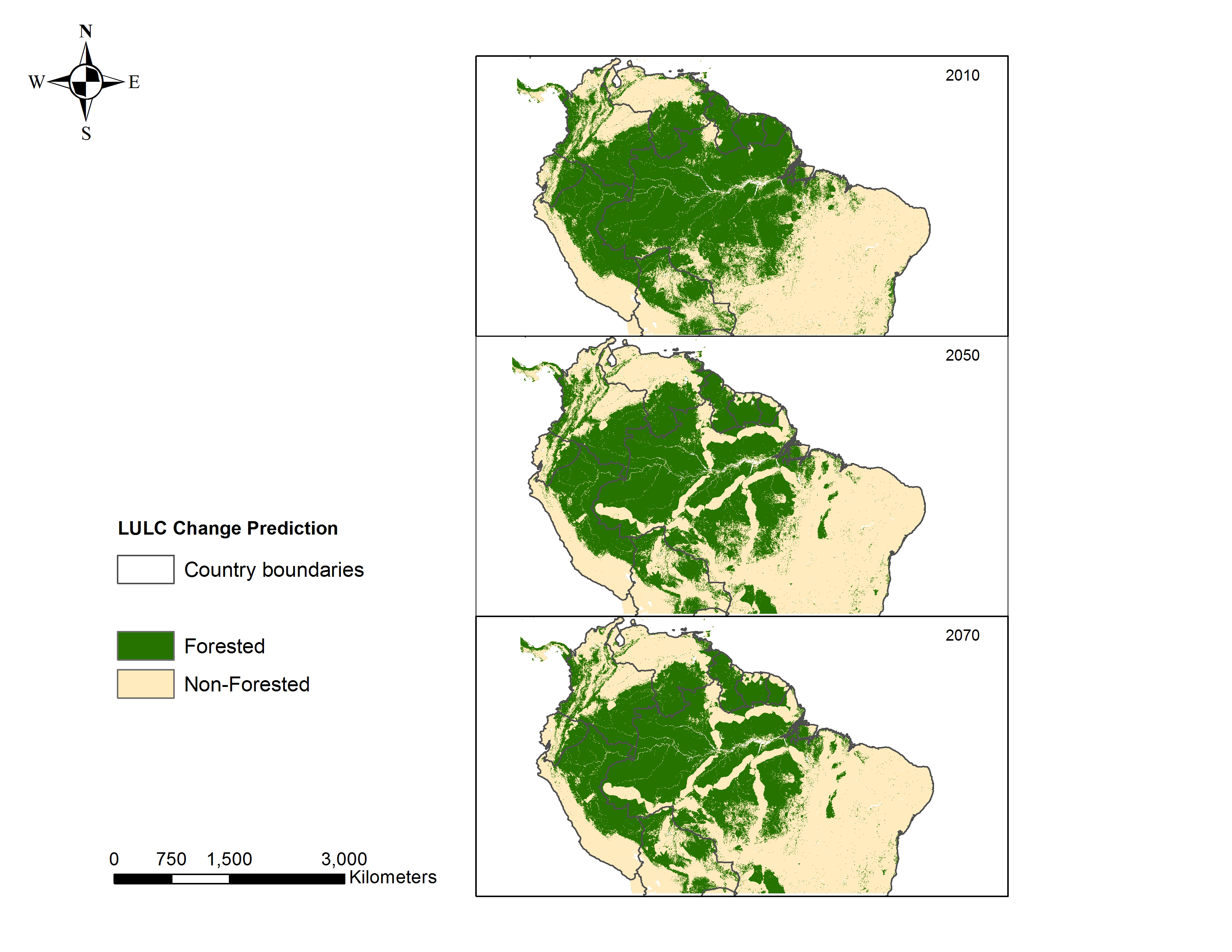

Supplement: Additional file 2: — Projected LULC changes obtained using LCM: 2010 (top panel), 2050 (middle panel), and 2070 (bottom panel). (TIFF 24675 kb) [file 13071_2015_1033_MOESM2_ESM.tif]

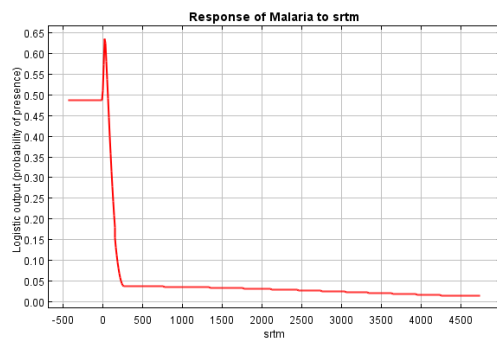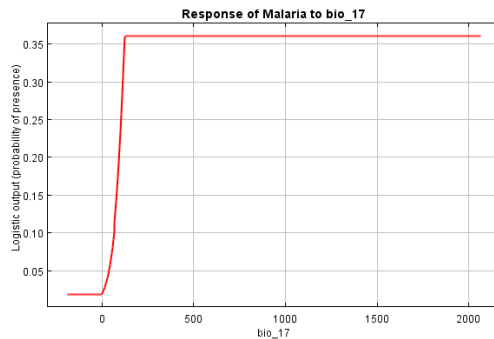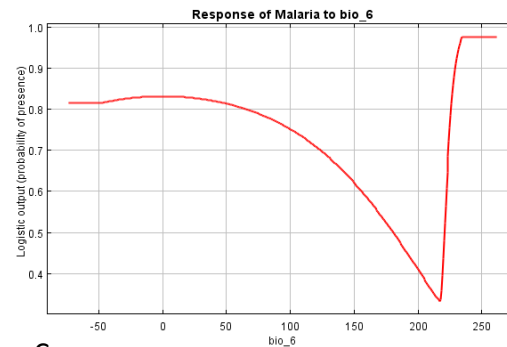

A

B

C

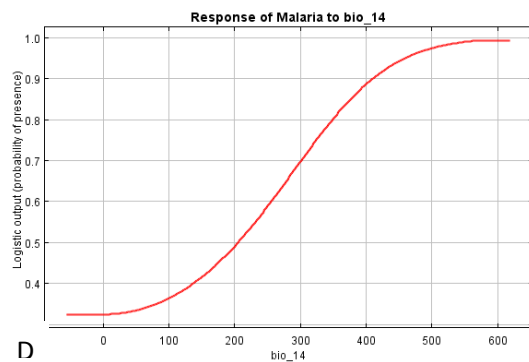

D

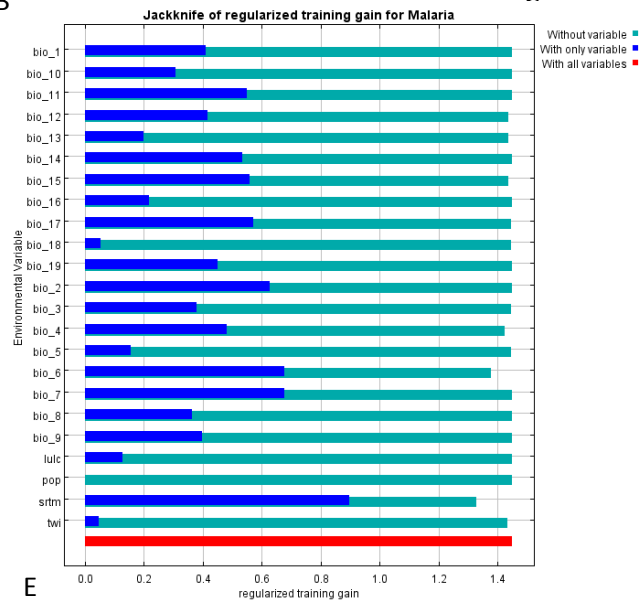

E

Supplement: Additional file 3: — Response curves of Malaria model to: (A) Elevation (srtm); (B) Precipitation of the driest quarter (bio 17); (C) Mean temperature of the coldest month (bio 6); (D) Precipitation of the driest month (bio 14); and (E) Jacknife of variable importance (Red bar shows the gain when all variables are used. The light blue bar shows the gain when a specific variable is excluded from analysis, a lower gain indicating that the specific variable has more information not contained in other variables. The dark blue bar indicates gain when the specific variable is used in isolation). (PDF 257 kb) [file 13071_2015_1033_MOESM3_ESM.pdf]

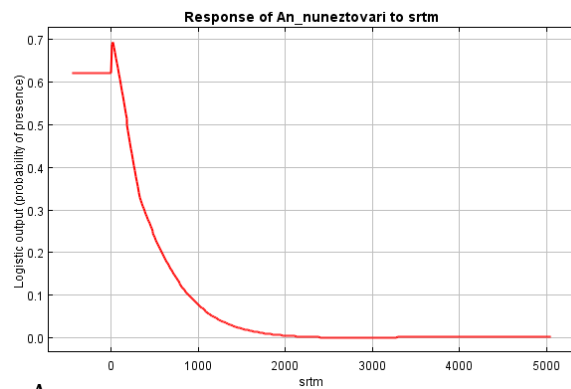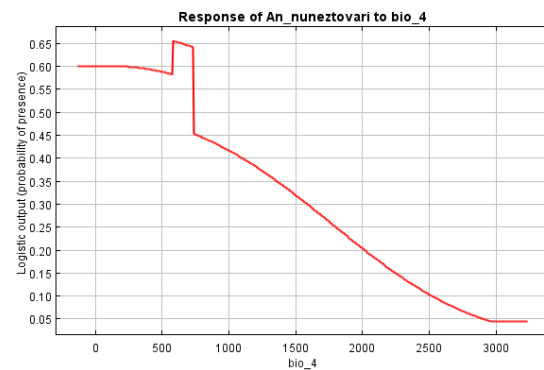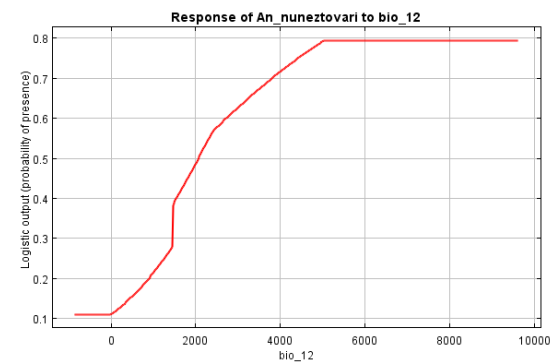

A

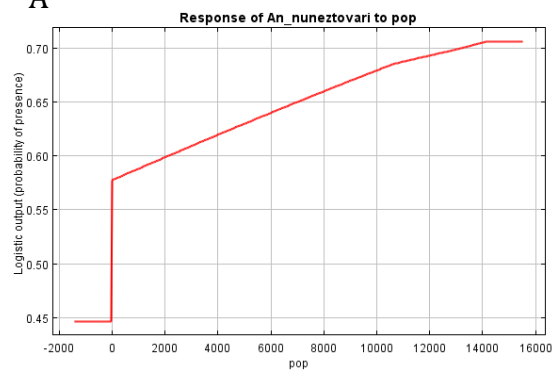

B

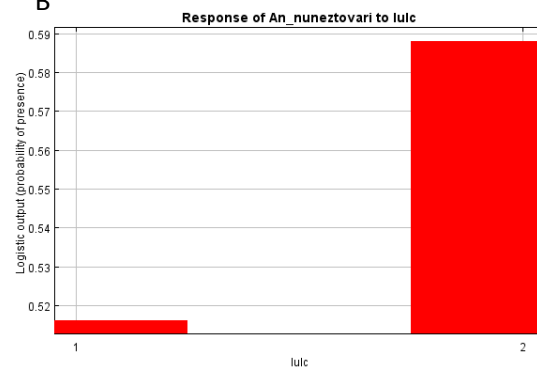

E

C

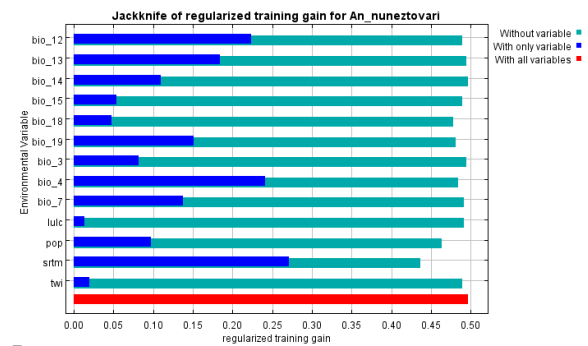

F

D

Supplement: Additional file 4: — Response curves of An. nuneztovari model to: (A) Elevation (srtm); (B) Temperature seasonality (bio 4); (C) Annual precipitation (bio 12); (D) Population; (E) LULC; and (F) Jacknife of variable importance (Red bar shows the gain when all variables are used. The light blue bar shows the gain when a specific variable is excluded from analysis, a lower gain indicating that the specific variable has more information not contained in other variables. The dark blue bar indicates gain when the specific variable is used in isolation) (PDF 258 kb) [file 13071_2015_1033_MOESM4_ESM.pdf]

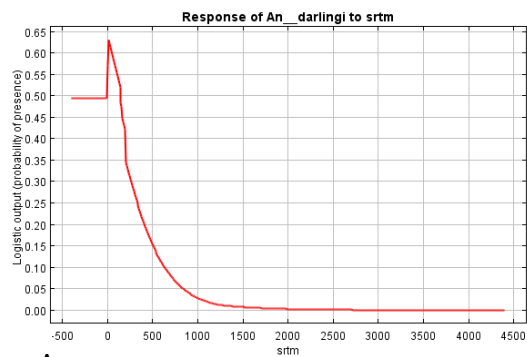

A

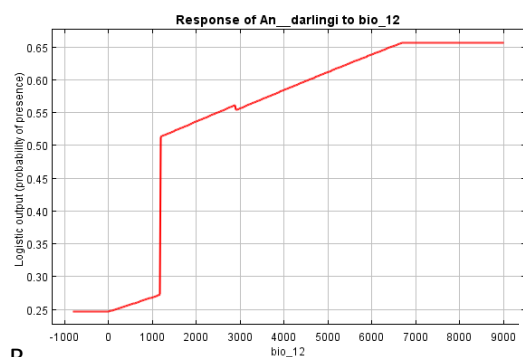

B

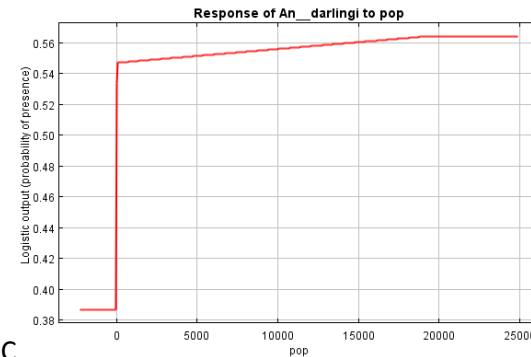

C

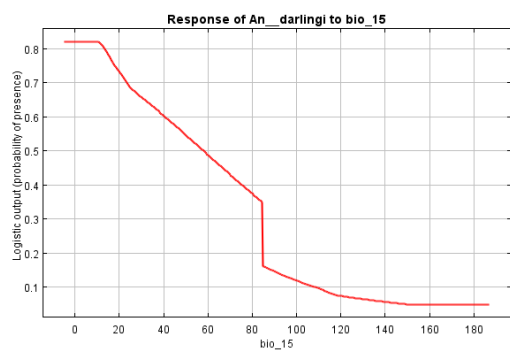

D

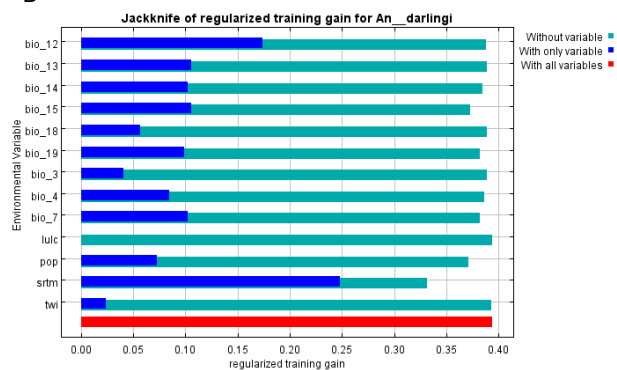

E

Supplement: Additional file 5: — Response curves of An. darlingi model to: (A) Elevation (srtm); (B) Annual precipitation (bio 12); (C) Population; (D) Precipitation seasonality (bio 15); and (E) Jacknife of variable importance (Red bar shows the gain when all variables are used. The light blue bar shows the gain when a specific variable is excluded from analysis, a lower gain indicating that the specific variable has more information not contained in other variables. The dark blue bar indicates gain when the specific variable is used in isolation). (PDF 255 kb) [file 13071_2015_1033_MOESM5_ESM.pdf]

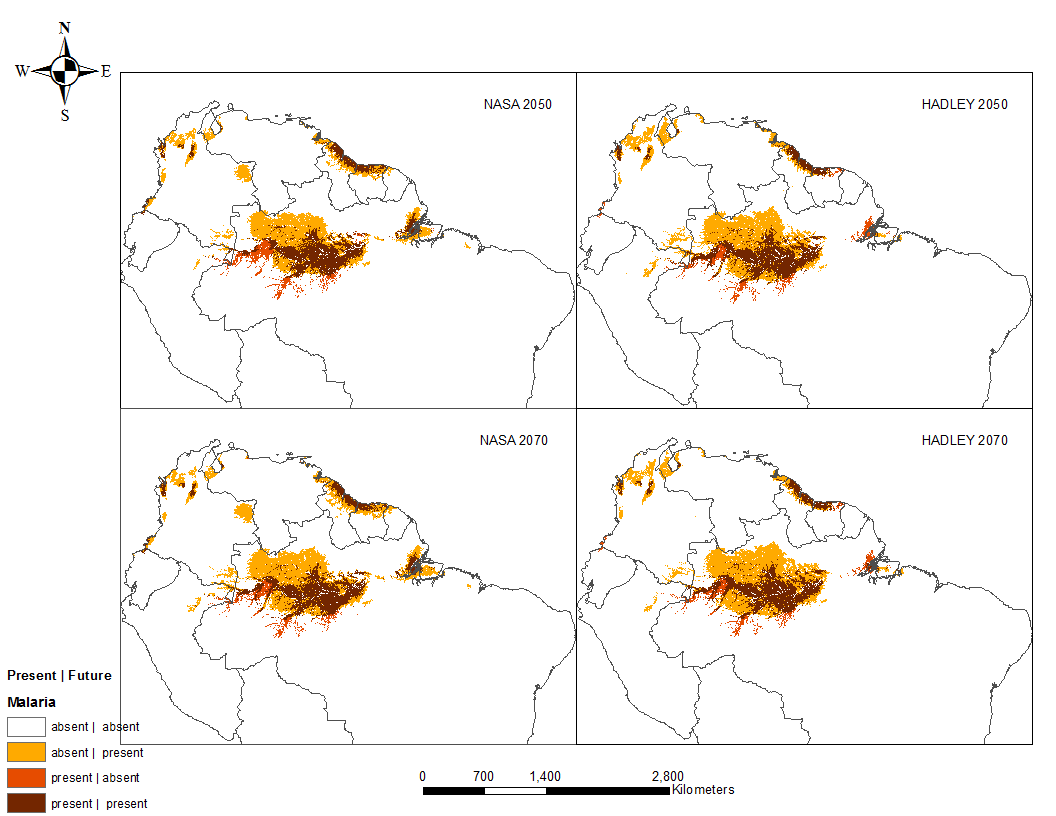

Supplement: Additional file 7: — Cross-tabulation of present and future distribution to show likely shifts in Malaria habitat suitability: NASA 2050 (top left), 2070 (bottom left) and Hadley 2050 (top right), 2070 (bottom right). (TIFF 2525 kb) [file 13071_2015_1033_MOESM7_ESM.tif]

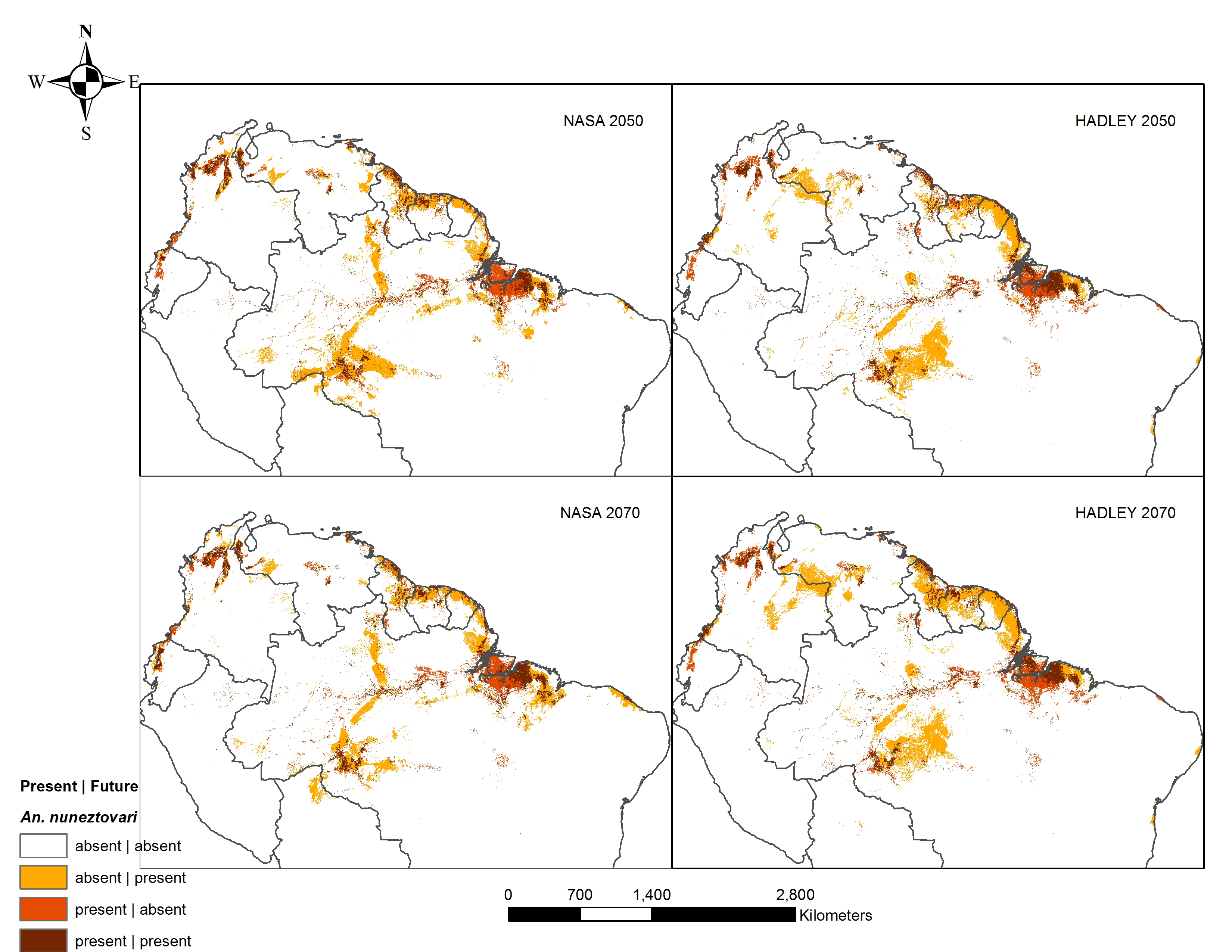

Supplement: Additional file 8: — Cross-tabulation of present and future distribution to show likely shifts in An. nuneztovari s.l. habitat suitability: NASA 2050 (top left), 2070 (bottom left) and Hadley 2050 (top right), 2070 (bottom right). (TIFF 24676 kb) [file 13071_2015_1033_MOESM8_ESM.tif]

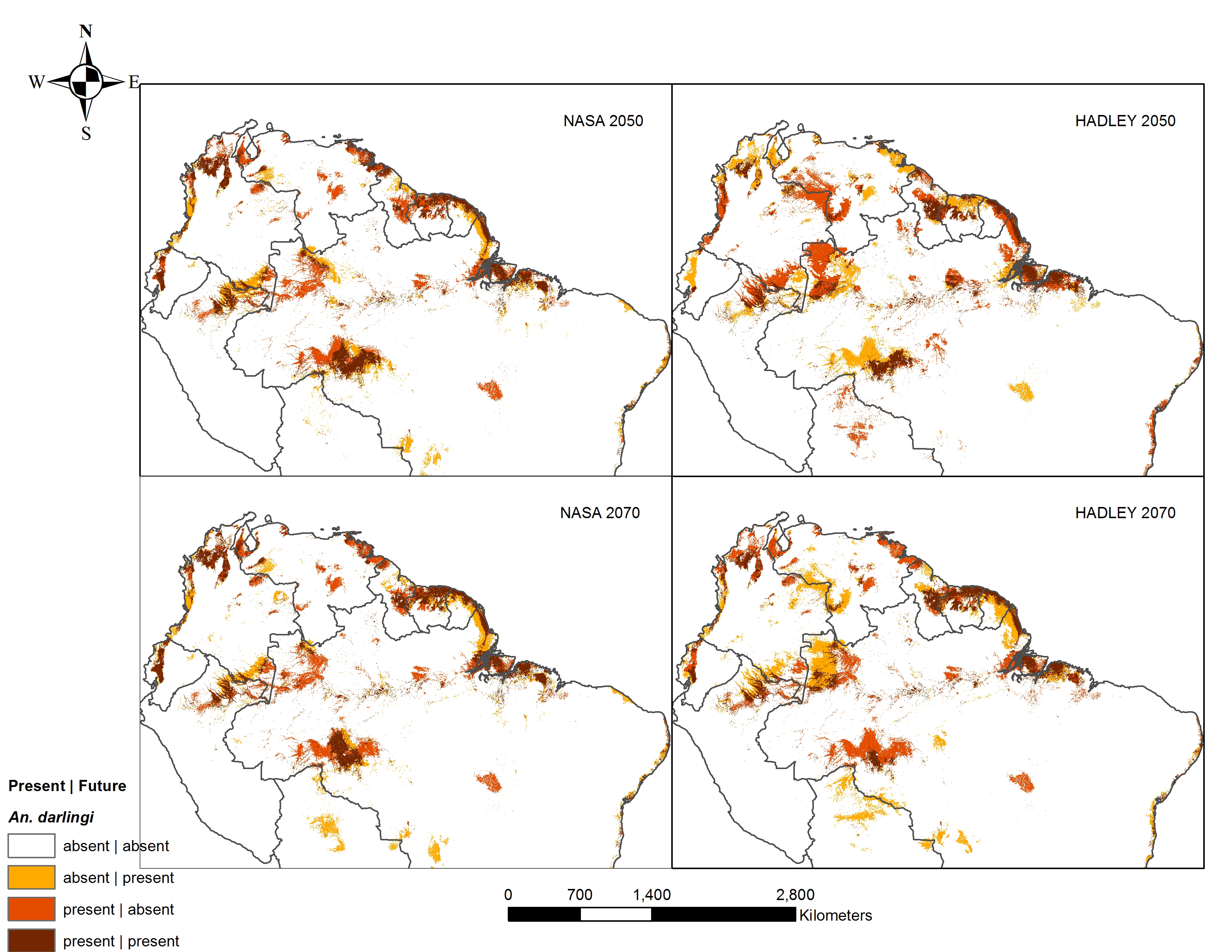

Supplement: Additional file 9: — Cross-tabulation of present and future distribution to show likely shifts in An. darlingi habitat suitability: NASA 2050 (top left), 2070 (bottom left) and Hadley 2050 (top right), 2070 (bottom right). (TIFF 24677 kb) [file 13071_2015_1033_MOESM9_ESM.tif]
